# Brief Report on screening data from a randomized controlled trial of in-hospital buprenorphine initiation strategies

**DOI:** 10.21203/rs.3.rs-8695127/v1

**Published:** 2026-05-21

**Authors:** Abigail Haber, Guillermo Sanchez-Fat, Michele Buonora, Annika Sabado, Megan Ghiroli, Yuting Deng, Laila Khalid, Kristine E. Torres-Lockhart, Marilyn Reyes, Kelly S Ramsey, Joanna L. Starrels, Aaron D. Fox

**Affiliations:** Albert Einstein College of Medicine; Albert Einstein College of Medicine; Albert Einstein College of Medicine; Albert Einstein College of Medicine; Albert Einstein College of Medicine; Albert Einstein College of Medicine; Albert Einstein College of Medicine; Albert Einstein College of Medicine; Albert Einstein College of Medicine; Albert Einstein College of Medicine; Albert Einstein College of Medicine; Albert Einstein College of Medicine

**Keywords:** opioid use disorder, chronic pain, addiction treatment, artificial intelligence

## Abstract

**Objectives:**

Buprenorphine (BUP) is effective for treating opioid use disorder and chronic pain (CP); however, there are missed opportunities for initiation during hospitalization. We conducted a clinical trial of in-hospital BUP initiation strategies and reviewed electronic health record data (EHR) to identify potentially eligible patients for BUP treatment. We present trial screening data to analyze the efficiency of EHR surveillance to identify BUP-eligible patients.

**Methods:**

Recruitment occurred between 10/2022 to 10/2024 at two urban teaching hospitals. An automated EHR algorithm generated a daily list of patients with likely opioid misuse or OUD. Preliminary manual review of the EHR algorithm was conducted by staff, who then contacted potentially eligible patients and their physicians before assessing for trial eligibility and BUP treatment. If patients were not assessed, research assistants use pre-specified categories to document reasons why.

**Results:**

The algorithm produced 9140 records for screening and 8534 (93%) were excluded based on preliminary manual review. Of 434 potentially eligible patients, only 43 agreed to be assessed for the trial. Of the 43 patients, 29 were eligible and 23 enrolled in the trial and started BUP, yielding a 5% initiation rate among the pool of potentially eligible patients.

**Conclusions:**

While the automated EHR algorithm identified patients potentially eligible for BUP, substantial manual review was required. Improving algorithm precision, for example with artificial intelligence applications, could better identify BUP-eligible patients; nonetheless, our experience suggests that patient concerns and potential stigma regarding BUP present as ongoing obstacles to BUP uptake.

## Introduction

Buprenorphine (BUP), a safe and effective treatment for opioid use disorder (OUD) and chronic pain (CP), has similar analgesic efficacy and lower overdose risk when compared to full agonist opioids (FAOs). BUP has become a preferred opioid to treat OUD and/or CP; however, BUP prescriptions have plateaued in the US despite increases in OUD prevalence. The frequency at which people with OUD and CP are hospitalized warrants additional research on starting BUP treatment during hospitalization.

To identify hospitalized patients who could potentially benefit from BUP, we developed an automated algorithm using electronic health record (EHR) data to identify patients with likely opioid misuse or OUD, and procedures for contacting patients and inpatient medical providers. The research was part of a clinical trial examining strategies for BUP initiation during hospitalization for patients with OUD or opioid misuse and CP (NCT 05118204). This analysis used trial screening data to examine the efficiency of EMR surveillance in identifying BUP-eligible patients.

## Methods

### Setting & Participants:

Recruitment occurred between 10/2022 to 10/2024 at two teaching hospitals in an urban area with the same EHR (EPIC, Verona, WI). All hospitalized patients identified by the algorithm and screened for the clinical trial were included here. Table 1 shows eligibility criteria for the trial.

### Data Collection & Analysis:

Screening had 4 stages. First, an automated EHR algorithm produced daily reports identifying hospitalized patients with opioid misuse with ≥ 1 of these criteria (Table 1):

(1) ≥ 1 of 12 drug-related terms (e.g. “IVDU”) in EHR notes; (2) opioid positive urine toxicology; (3) ≥ 1 relevant ICD-10 code; (4) prior medication for OUD prescription; or (5) treatment or assessment for opioid withdrawal. Though CP was an inclusion criterion for the trial, we did not include ICD-10 codes in the EHR algorithm because of our specific CP definition (see below). Second, a research assistant (RA) manually reviewed each record to determine preliminary trial eligibility, including the presence of CP. During manual review, CP was defined by the presence of the term ‘chronic pain’ in the EMR and/or documentation in clinical notes indicating pain duration of ≥ 3 months. An addiction specialist then rereviewed selected records to exclude clinically unstable patients (e.g., hospitalized for sepsis) and confirm that BUP was clinically warranted. Third, an RA approached patients and attending physicians to discuss the trial and BUP treatment. Finally, if patients were interested in BUP, the RA and addiction specialist assessed them for trial eligibility and BUP treatment. RAs confirmed CP during screening, with CP defined as pain that is chronic (on most or all days for ≥ 3 months) and at least moderate intensity and interference (score ≥ 4 on Pain, Enjoyment of Life and General Activity Scale [PEG].) RAs documented reasons why patients were not assessed based on pre-specified categories.

We report counts for patients who: were potentially eligible for the trial, could and could not be approached, agreed to be assessed, and reasons potentially eligible patients were not assessed for BUP.

## Results

**Figure 1:**
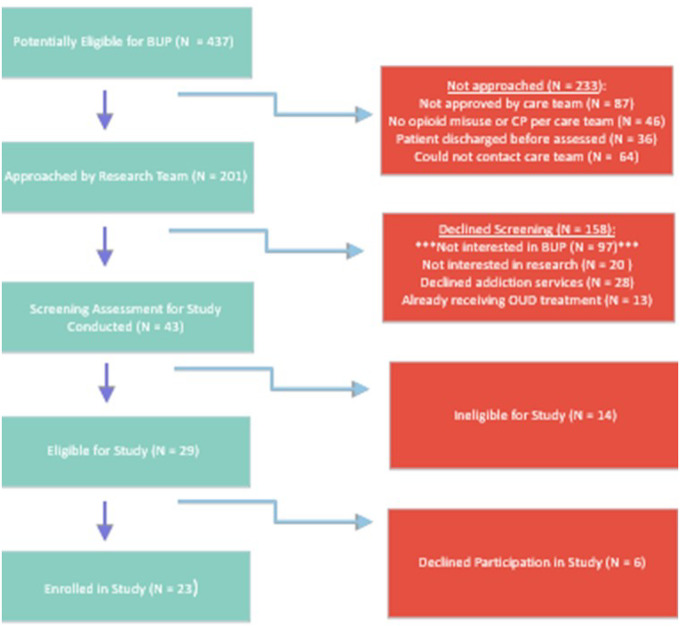
Hospitalized Patients with CP and OUD or opioid misuse potentially eligible for a BUP treatment clinical trial ^1^The definitions for the abbreviations used in Figure 1 are as follows: CP = chronic pain OUD = opioid use disorder BUP = buprenorphine

The EHR algorithm produced 9140 records, with 8534 excluded based on preliminary manual review. The most common reasons were: no identified opioid misuse (misidentification) (43%); receiving OUD medication before admission (21%); or study-specific exclusions (e.g. documented hypoxia) (16%). Of the 434 patients eligible after preliminary manual review and confirmation by the addiction specialist, 201 were successfully approached. Of these 201, many declined any addiction services (14%), or buprenorphine specifically (48%). Of the 43 that agreed to be assessed, 29 were eligible and 23 enrolled in the study and started BUP. Thus, of 434 hospitalized patients that were potentially eligible, 5% started BUP (Fig. 1).

## Discussion

In a hospital-based BUP trial, we had mixed results from a staged screening process intended to identify patients with opioid misuse or OUD and CP. Through EHR algorithm identification and manual review, we identified 434 hospitalized patients who potentially could have benefitted from BUP. Although designed as a research tool only, the algorithm misidentified 43% of records as having opioid misuse and requires some scrutiny. Once potentially eligible patients were identified, challenges in considering BUP treatment were the high acuity of illness and difficulty approaching patients before hospital discharge, which reflects the difficulty of treating chronic conditions like OUD or CP during acute hospitalizations.

While hospitalization can be a “touchpoint” for care, reaching patients with CP and likely opioid misuse or OUD to offer BUP can be difficult.^6^ Research supports BUP initiation in emergency departments and by inpatient addiction services, but hospitalists are often focused on treating other acute medical problems rather than OUD. Our study focused on patients with OUD or opioid misuse and CP, and transitioning from FAOs to BUP for CP during hospitalization also involves challenges. An addiction specialist screened records to determine whether BUP was clinically indicated, but over half of patients declined addiction services or BUP treatment specifically. Therefore, while hospital-based clinicians should be prepared to prescribe BUP, the high acuity environment and potential patient concerns about BUP may impede uptake.

Our automated algorithm deserves comment. Others have designed screening algorithms to identify OUD. One study developed a 1288-term dictionary for mentions of opioid addiction or misuse, and a natural language processing (NLP) system to find these mentions in unstructured text. Two other studies used machine learning to develop a continuous measure of problematic opioid risk. In our study, adopting an NLP system to distinguish affirmative mentions from those negated or qualified (e.g. “illicit drugs” vs “denies illicit drugs”), could have proven useful, as we received many “false positives” from our algorithm.^6^ Using these tools to identify hospitalized patients for addiction medicine consultation could be beneficial.

## Conclusion

While the EMR algorithm identified some potentially BUP-eligible patients, our screening and chart review process could use refinement. Artificial intelligence applications could improve algorithm precision and better identify BUP-eligible patients; however, patient concerns, and potential stigma regarding BUP treatment, could be ongoing challenges to BUP uptake. Better screening methods and interventions to improve buy-in from patients and providers could still increase BUP uptake during hospitalization.

## Figures and Tables

**Table 1:** Inclusion Criteria for Study Algorithm Patients meet inclusion criteria if ≥ 1 criterion is met.

Opioid-related indicators, and/or additional evidence of OUD (≥ 1 criterion)
v Have prior diagnosis for OUD within 1 year of the current hospitalization (ICD-10Dx codes F11.x,T40.x or Z79.891)
v Have positive drug test during hospitalization (**URINE TOXICOLOGY or DRUG SCREEN)** excluding
o *AMPHETAMINE*
o *BARBITURATE*
o *BENZODIAZEPINE*
o *CANNABINOID*
o *COCAINE*
o *MARIJUANA*
o *PHENCYCLIDINE*
v Have the following phrases in the notes related to hospitalization
o ADDICTION CONSULTATION
o opioid addiction
o opioid abuse
o methadone maintenance
o opioid dependence
o polysubstance
o injection drug
o illicit drugs (but not phrases like “denies illicit drugs”, “never done illicit drugs”, “does not use any illicit drugs”, etc.)
o IVDU
o drug seeking
o stoned
o drugged

## References

[1] Butrans (buprenorphine) Transdermal System [Package Insert]. Stamford, CT: Purdue Pharma L.P. 2014.

[R2] Aiyer R, Gulati A, Gungor S, Bhatia A, Mehta N. Treatment of Chronic Pain With Various Buprenorphine Formulations: A Systematic Review of Clinical Studies. Anesth Analg. 2018;127(2):529–538. doi:10.1213/ANE.0000000000002718 29239947

[R3] Bart G. Maintenance medication for opiate addiction: the foundation of recovery. J Addict Dis. 2012;31(3):207–225. doi:10.1080/10550887.2012.694598 22873183 PMC3411273

[R4] Gudin J, Fudin J. A Narrative Pharmacological Review of Buprenorphine: A Unique Opioid for the Treatment of Chronic Pain. Pain Ther. 2020;9(1):41–54. doi:10.1007/s40122-019-00143-6 31994020 PMC7203271

[R5] Jones JD, Sullivan MA, Manubay J, Vosburg SK, Comer SD. The subjective, reinforcing, and analgesic effects of oxycodone in patients with chronic, non-malignant pain who are maintained on sublingual buprenorphine/naloxone. Neuropsychopharmacology. 2011;36(2):411–422. doi:10.1038/npp.2010.172 20980992 PMC3055661

[R6] Weimer MB, Buonora MJ, Hajduk AM, Intervention for hospitalized people with chronic pain and elevated risk for opioid-related harm: a pilot randomized controlled trial. J Hosp Med. 2025; 1–8. doi:10.1002/jhm.70066 PMC12354179 40271961

[R7] Hayes BT, Sanchez Fat G, Torres-Lockhart K, Low-Dose Buprenorphine Initiation for Hospitalized Patients With Chronic Pain and Opioid Use Disorder or Opioid Misuse: Protocol for an Open-Label, Parallel-Group, Effectiveness-Implementation Randomized Controlled Trial. Substance Use & Addiction Journal. 2024;46(1):184–191. doi:10.1177/29767342241263221 39068540 PMC11652261

[R8] Larochelle MR, Bernstein R, Bernson D, Touchpoints - Opportunities to predict and prevent opioid overdose: A cohort study. Drug Alcohol Depend. 2019;204:107537. doi:10.1016/j.drugalcdep.2019.06.039 31521956 PMC7020606

[R9] Carrell DS, Cronkite D, Palmer RE, Using natural language processing to identify problem usage of prescription opioids. Int J Med Inform. 2015;84(12):1057–1064. doi:10.1016/j.ijmedinf.2015.09.002 26456569

[R10] Afshar M, Joyce C, Dligach D, Subtypes in patients with opioid misuse: A prognostic enrichment strategy using electronic health record data in hospitalized patients. PLoS One. 2019;14(7):e0219717. Published 2019 Jul 16. doi:10.1371/journal.pone.0219717 31310611 PMC6634397

[R11] Sharma B, Dligach D, Swope K, Publicly available machine learning models for identifying opioid misuse from the clinical notes of hospitalized patients. BMC Med Inform Decis Mak. 2020;20(1):79. Published 2020 Apr 29. doi:10.1186/s12911-020-1099-y 32349766 PMC7191715

